# Next-generation phylogenomics

**DOI:** 10.1186/1745-6150-8-3

**Published:** 2013-01-22

**Authors:** Cheong Xin Chan, Mark A Ragan

**Affiliations:** 1 Institute for Molecular Bioscience, and ARC Centre of Excellence in Bioinformatics, The University of Queensland, Brisbane, QLD, 4072, Australia

**Keywords:** Phylogenomics, Multiple sequence alignment, Alignment-free methods, *k*-mers, Homology signal

## Abstract

**Abstract:**

Thanks to advances in next-generation technologies, genome sequences are now being generated at breadth (*e.g.* across environments) and depth (thousands of closely related strains, individuals or samples) unimaginable only a few years ago. *Phylogenomics* – the study of evolutionary relationships based on comparative analysis of genome-scale data – has so far been developed as industrial-scale molecular phylogenetics, proceeding in the two classical steps: multiple alignment of homologous sequences, followed by inference of a tree (or multiple trees). However, the algorithms typically employed for these steps scale poorly with number of sequences, such that for an increasing number of problems, high-quality phylogenomic analysis is (or soon will be) computationally infeasible. Moreover, next-generation data are often incomplete and error-prone, and analysis may be further complicated by genome rearrangement, gene fusion and deletion, lateral genetic transfer, and transcript variation. Here we argue that next-generation data require next-generation phylogenomics, including so-called *alignment-free* approaches.

**Reviewers:**

Reviewed by Mr Alexander Panchin (nominated by Dr Mikhail Gelfand), Dr Eugene Koonin and Prof Peter Gogarten. For the full reviews, please go to the Reviewers’ comments section.

## Background

Next-generation sequencing technologies are yielding genome-scale data in immense quantities: genomes and transcriptomes of viruses, bacteria, archaea and eukaryotes; single-cell isolates and clonal cultures; diverse cell types under normal, stress and disease conditions; meta-genomes and meta-transcriptomes. With a steady decline in costs as new technologies are developed and/or refined, sequencing projects nowadays are not only taxonomically broad but increasingly deep, *e.g.* the 1001 Genomes Project for *Arabidopsis* (1001genomes.org) and ~1000 isolates of *Staphylococcus aureus* sequence type 239 (http://www.ebi.ac.uk/ena/). Plans are afoot to sequence 100,000 human genomes (personalgenomes.org) and 100,000 food-borne pathogens (100kgenome.vetmed.ucdavis.edu). Next-generation data offer particular promise in the study of population genomics and variation, and of the genetic mechanisms underlying how organisms respond to their environments.

While prospects have never been brighter for data generation, genome projects may be limited by the supply of human and computational power for data analysis. Assembly (de-replication of overlapping reads to yield a single contiguous sequence) is computationally expensive even for a single large genome, and approaches impossibility against the backdrop of noise (*e.g.* sequencing errors, contaminating DNA), regions of low information content (repeats, telomeres) and among-individual heterogeneity. Given the technologies and services currently on offer, advanced centres (*e.g.* BGI in China and the Joint Genome Institute in USA) are simply sequencing to high coverage, even for prokaryotes, in the most-ambitious projects (*e.g.* Genomic Encyclopedia of Bacteria and Archaea: jgi.doe.gov/programs/GEBA/). The resulting unfinished data, replete with un-joined contigs, ambiguous assemblies and erroneous base calls, will be noisier, yet far more abundant, than the tidy closed circles that up to now have been iconic of microbial genomics.

### Phylogenomics in the new era

Phylogenomics, the study of evolutionary relationships based on comparative analysis of genome-scale data, is indispensible in assessing diverse biological hypotheses, *e.g.* the distribution and spread of bacterial pathogenicity, the convergence or divergence of gene function, the origin of organelles, or resolution of the tree (or network) of life. Relationships among taxa are inferred based on homology (inheritance from a common ancestor, commonly observed as patterns of sequence similarity) across entire genomes, whether in a comparative gene-by-gene [1,2], concatenated multi-gene [3,4] or whole-genome approach [5]. Genomes of economically or medically important species and of “model” organisms (*Arabidopsis*, *Drosophila*) were the first to be sequenced and until very recently predominated in public databases, although as cost per base has decreased, other factors (*e.g.* phyletic position, role in the environment) have begun to drive sequencing decisions. Where genome data are unavailable (*e.g.* too technically challenging due to compositional bias, low complexity, long repeats or polyploidy), it is not uncommon to utilise transcriptome data in phylogenomic analysis [6-8], at some cost of lost information (weakly or differentially expressed genes, partial transcripts). Even so, studies adopting these “conventional” phylogenomic approaches (*e.g.*[2,3,9,10]) have yielded unprecedented insight into physiology and evolution, and have generated novel hypotheses for future exploration [11-13].

These approaches, however, are not without limitations, especially when evolutionary histories are complicated [14,15]. Like gene-by-gene phylogenetics, phylogenomics must accommodate (stochastic) substitution-rate variation and biases across sites and lineages, incomplete taxon sampling and, especially for prokaryotes and microbial eukaryotes, lateral genetic transfer [6,10,16-18]. Increasingly it must also deal with variable sequence quality (including mis-assembly), copy-number variation, recombination, gene fusion and gene deletion. Eukaryotes, the fastest-growing market share, add further phylogenomic challenges including diverse chromosomal inheritance patterns, partial or whole-genome duplication, expansion and contraction of gene families, alternative splicing and other forms of transcriptional variation, non-protein-coding genes, mobile elements, and epigenetic modifications [14,19,20].

A comprehensive, sustainable strategy for phylogenomics should therefore transcend gene boundaries – whatever those may be – while capturing, or at least not being led astray by, the complex dynamics playing out both within genes (transcriptional variation) and in the vast intergenic regions. Current best-practice phylogenomics cannot do this adequately or at the necessary scale.

### Multiple sequence alignment and its limitations

**Figure 1:**
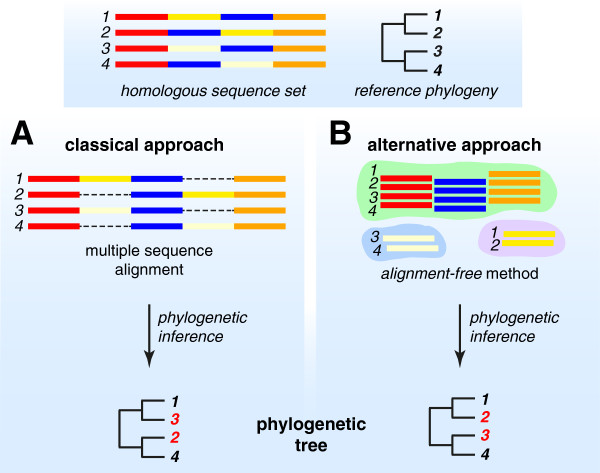
**Simplified workflow of phylogenomic approaches.** Workflow is shown for **(A)** the classical approach based on multiple sequence alignment, and **(B)** an alternative approach based on alignment-free methods, for a simple analysis example of homologous sequences *1*, *2*, *3* and *4*, with a known phylogeny as a reference (shown on top). Sequence fragments that share the same ancestry across all four sequences (*i.e.* are highly similar among one another) are shown in the same colour (red, blue, yellow and orange regions in each sequence). In this example, the yellow and blue regions of sequences *2* and *4* have undergone rearrangement relative to *1* and *3*. The dark yellow (in *1* and *2*) and light yellow (in *3* and *4*) regions are similar to each other. While the classical approach based on multiple sequence alignment (gaps introduced as dashed lines) yields an inaccurate phylogeny, the alternative alignment-free approach (grouping of sub-sequences) is not affected by the sequence rearrangement in *2* and *4*, and yields the correct phylogeny. The difference between the two resulting phylogenetic trees is highlighted in red.

Multiple sequence alignment (MSA) has long been a *sine qua non* in phylogenetics [21]. The aim of MSA is to arrange sequence regions relative to each other in a way that presents (to the tree-inference software) the best available hypothesis of homology at each and every position. Even when these positions have maintained their contiguity and relative order through evolutionary history, reconstructing this history requires assumptions about substitution models and uniformity of process across sites and branches, and involves the application of memory-intensive algorithms and heuristics [22,23]. Local structural variation can lead to “gappy” alignments that degrade resolution and bias phylogenetic inference (Figure 1A). Some of the processes mentioned above – recombination, duplication, gain and loss – play out *within* genes as well, yielding regions that can be aligned only ambiguously, or not at all. Given the heuristic nature of key steps in standard phylogenomic workflows, the relevance of alignment scores to homology can be difficult to assess statistically [24]. All of these issues are intensified at full-genome scale, and few are resolvable by increased computing power or better substitution models.

MSA of highly divergent homologous sequences, *e.g.* proteolipids of ATPase [25] or aquaporins in plants [26], is known to be problematic. A number of approaches, while not entirely independent from MSA, have been adopted to address the limitations of MSA. For example, Thorne and Kishino [27] estimated pairwise evolutionary distance based on insertion-deletion and amino acid replacements, instead of sequence identity. Other strategies include a divide-and-conquer approach [28], in which MSA was performed on closely related subsets of sequences, then the information from these aligned subsets was used to guide and improve the overall MSA of the sequences, on which phylogenetic inference was based. Similarly, a phylogenetic tree can be inferred from each of these aligned subsets before they are combined, by consensus, into a final tree [29]. The divide-and-conquer strategy has also been applied to classify protein sequences based on conserved profiles, with MSA of proteins guided by multiple-profile alignments [30]. These approaches, although independent of MSA in the conventional sense, still assume full-length contiguity of the sequences under comparison. We argue here that next-generation phylogenomics must aspire to become more fully independent of multiple sequence alignment, while capturing as much homology signal as possible in the face of genome dynamics including lateral genetic transfer.

### Alignment-free methods

Approaches based on exact sub-sequences of defined (but typically short) length, known variously as words, *k*-mers or *n*-grams, offer an interesting alternative to MSA. A *k*-mer can be treated as a unit of information. This motivates so-called *alignment-free* approaches in which *k*-mers are extracted and their counts or frequency distributions (*i.e. k*-mer spectra) are computed; sequencing errors can appear as unexpected *k*-mers, while gene-regulatory regions, repetitive elements or laterally transferred regions can exhibit unexpected *k*-mer spectra (see [31] for a review).

In the same way, individual *k*-mers can be viewed as embodying parts of the homology signal in a sequence. If *k*-mers bearing enough unbiased signal can be extracted, statistically based comparisons of *k*-mer spectra can be used to infer phylogenetic relationships or map genetic transfer [32,33]. Studies based on simulated data suggest that trees based on pairwise distances computed from *k*-mer statistics can, under certain circumstances at least, be more accurate than those based on MSA [34]. By decoupling homology signal from sequence contiguity beyond word length, *k*-mer methods simply avoid the computational complexity of MSA while capturing signal otherwise lost to gappiness, recombination or shuffling (Figure 1B). In learning to extend *k*-mer approaches to datasets containing mis- or un-assembled contigs, overlapping transcripts, gene fragments or low-quality sequences, there is a great opportunity to draw on experience in fields less familiar to (and less well-mined by) evolutionary biologists, including signal transmission and information retrieval [35]. Table 1 compares key features of phylogenomic approaches based on MSA and alignment-free methods.

**Table 1:** Comparison of key features between phylogenomic approaches based on multiple sequence alignment and alignment-free approaches

**Approach based on multiple sequence alignment**	**Approach based on alignment-free methods**
Assumes contiguity (with gaps) of homologous regions	Does not assume contiguity of homologous regions
Based on all possible pairwise comparisons of whole sequences; computationally expensive	Based on occurrences of sub-sequences; computationally inexpensive, can be memory-intensive
Well-established and well-studied approach in phylogenomics	Application in phylogenomics limited; requires further testing for robustness and scalability
More dependent on substitution/evolutionary models	Less dependent on substitution/evolutionary models
More sensitive to stochastic sequence variation, recombination, lateral genetic transfer, rate heterogeneity and sequences of varied lengths, especially when similarity lies in the “twilight zone”	Less sensitive to stochastic sequence variation, recombination, lateral genetic transfer, rate heterogeneity and sequences of varied lengths
Best practice uses inference algorithms with complexity at least *O*(*n*^2^); less time-efficient	Inference algorithms typically *O*(*n*^2^) or less; more time-efficient
Heuristic solutions; statistical significance of how alignment scores relate to homology is difficult to assess	Exact solutions; statistical significance of the sequence distances (and degree of similarity) can be readily assessed

In one class of alignment-free approach, relatedness of two sequences is based on the number and value of sub-sequences (*e.g. k*-mers) they share in common. This measure can be transformed (*e.g. via* logarithmic representation of the geometric mean) to estimate an evolutionary distance. A matrix of pairwise distances built on these statistics for a set of sequences can then be used to reconstruct a phylogeny. The measure of relatedness can be based on the frequency (number of occurrences) of *k*-mers, or can also take into account their relative positions within the sequences. Correlation among the common *k*-mer sets, sometimes adjusted by the value of *k* (length) relative to whole sequence, can be incorporated as an up- or down-weighting factor. This measure can alternatively be normalised by the probability at which corresponding *k*-mers occur in the sequences, or extended to include imperfect *k*-mer matches [36]. No models of sequence change are explicitly invoked. Alignment-free generation of the distance matrix is computationally faster than MSA, although the memory requirement can be substantial. Quick and simple (yet well-behaved) algorithms such as neighbour-joining can then be applied to calculate the tree, rather than computationally complex methods such as maximum likelihood or Bayesian approaches. Alternative approaches not based on *k*-mer distances have also been put forward [32,37,38].

For each position in a query sequence, the length of the shortest unique substring that is absent in other (subject) sequences can be used to infer the relatedness among these sequences, and the positions at which a particular subject sequence is most similar to the query can be used to infer genetic transfer [33,39]. In addition, *k*-mers have been used to partition and classify metagenomic data based on compositional biases of genome sequences, such that sequences with a particular abundance distribution of *k*-mers are grouped together (see [40] for review).

### Discussion and conclusions

A key driver in phylogenomics is the improvement of existing phylogenetic algorithms so that we can infer, at large scale, phylogenetic relationships with minimal technical biases and greater computational efficiency. The use of heuristics in approximating a maximum likelihood approach has sped up the process of phylogenetic inference [41], although with some sacrifice in accuracy. Maximum likelihood requires specification of a (potentially unrealistic) evolutionary model according to which the sequences are assumed to have evolved. Bayesian inference requires specification of priors, which can be tricky without prior understanding of the data. While the speed and complexity of these approaches could be optimised and managed using heuristics or the divide-and-conquer strategy described above, we are limited by the drawbacks of MSA (Table 1).

Before we can dispense altogether with MSA the scalability, robustness and efficiency of *k*-mer statistics in genome-wide comparison need to be rigourously tested. Fundamental operations of sub-sequence extraction and indexing (algorithms, computation and memory usage) are a good place to start; but beyond those, problems previously encountered in engineering or data-mining may not map precisely to evolutionary biology. Every attempt should be made to carry over into next-generation phylogenomics the generally quadratic to cubic time-complexity of phylogenetic distance methods on trees [42] or networks [43]. Moreover a new, principled approach to data reduction will be necessary with the increasing depth imbalance of genome data. On the other hand, next-generation phylogenomics could allow the use of multiple data types (*e.g.* genome, transcriptome, proteome and/or metabolome) in a one-stop inference of evolutionary relationships, hybrid approaches (*e.g.* applying *k*-mer- and model-based methods for more and less similar sequences respectively), or functional inference based on *k*-mer spectra.

Like molecular phylogenetics in the 1970s, alignment-free phylogenomics has just entered a period of development, refinement and application. Major aims can be articulated – reconstructing complex biological scenarios efficiently and well, based on unprecedented volumes of new data and data types – although the best algorithmic paths to those aims remain to be discovered and explored. To the extent that these paths prove to be scalable and robust, next-generation phylogenomics may be alignment-free.

## Abbreviation

MSA: Multiple sequence alignment.

## Competing interests

The authors declare that they have no competing interests.

## Authors’ contributions

CXC and MAR conceived and wrote the manuscript. Both authors read and approved the final manuscript.

## Reviewers’ comments

Reviewer’s report 1: Mr Alexander Panchin, Institute of Information Transmission Problems, Russian Academy of Sciences (nominated by Dr Mikhail Gelfand, Russian Academy of Sciences)

The article “Next-generation phylogenomics” by Cheong Xin Chan and Mark A. Ragan addresses the idea of alignment-free methods for phylogenetic analysis using abundant next generation genome-wide data. Although there is hardly anything in the article I could disagree with, and the ideas expressed are sound, I am unsure if this article brings anything new to the table. The article is about next-generation phylogenomics, yet no new phylogenetic algorithms, applications, comparisons or phylogenetic trees are presented. In my opinion the article is a well written mini-review; however the value of this contribution for Biology Direct is questionable.

**Authors’ response:***We thank the reviewer for his comments. We wrote this Comment to encourage the research community to consider alternative approaches for phylogenomics in light of the recent (continuing) deluge of sequence data. A full research paper detailing how the alternatives work better than existing approaches is important, but is beyond the scope of this Comment. We have modified the text to incorporate a more-detailed discussion of standard phylogenetic approaches and the limitations of multiple sequence alignment (see also comments from the other reviewers below). In agreement with Reviewers 2 and 3, we believe this Comment is timely and appropriate for the readership of Biology Direct.*

Reviewer’s report 2: Dr Eugene V. Koonin, National Center for Biotechnology Information, NIH, USA

Review of “Next-generation phylogenomics” by Chan and Ragan.

This is a timely Comment, indeed. I agree with the authors in that we (as a community) should seriously think about next generation phylogenomics. Doing phylogenomics with the current methods on many thousands of genomes is simply not a possibility. At the same time, not making full use of the new wealth of genomic data is unthinkable as this is the surest way to new insights.

**Authors’ response:***We thank the reviewer for his endorsement of the issues we have raised.*

What I am less enthusiastic about, are the alignment-free methods the authors discuss, in particular the *k*-mer-based approaches. To my knowledge, the prospects of these particular methods are very limited. Certainly, alignment-free approaches are attractive but from an information-theoretical standpoint, I find it dubious that they promise much progress, at least when large phylogenetic depths are involved. At this juncture, I am more optimistic about clever algorithmic improvements on the “conventional” phylogenomic methods. The prime example is FastTree [41] that, in my experience, has changed the practice of phylogenetic analysis by combining the (nearly) full rigour of maximum likelihood with the speed of methods like neighbour joining. Although the MSA problem itself may be even more challenging, promising developments are appearing in this area as well, *e.g.*[30].

**Authors’ response:***We agree that better and/or faster phylogenetic techniques, such as FastTree (and other methods; see comments of Reviewer 3 below) are important for the field to progress as more data become available. These methods, largely based on maximum likelihood, require the (perhaps unrealistic) assumption of a model under which the sequences evolve, and are computationally expensive. FastTree, for instance, implements heuristics approximating maximum likelihood in restricting tree search space, and while faster, it is less accurate than the standard maximum likelihood methods. These methods are largely based on MSA, which implicitly assumes full-length sequence contiguity. As the reviewer points out, the problem of MSA itself is more challenging. We have now incorporated a discussion of other phylogenetic alternatives in the main text (see also the report of Reviewer 3 below). The application of alignment-free methods in large-scale phylogenomics is currently limited, and the scalability and robustness of these methods remain to be systematically investigated. This approach, however, represents an attractive strategy in handling key limitations of multiple sequence alignment (as summarised in* Table 1*). We argue here that next-generation phylogenomics should consider alignment-free methods as an alternative – but not the only one.*

Reviewer’s report 3: Prof J. Peter Gogarten (University of Connecticut, USA)

Chan and Ragan provide a concise review of the advantages of alignment free approaches in comparative genomics. They point out that the calculation of multiple sequence alignments often is unreliable and computationally expensive. They review alignment free approaches and provide examples of their usefulness. A more detailed discussion of techniques to detect horizontally transferred genes and to bin sequences from metagenomes based on compositional signals might have provided additional examples for the power of alignment free approaches already in widespread use today (*e.g.*, [44-46]).

**Authors’ response:***We thank the reviewer for his comments. We have now incorporated in the text a discussion of the use of alignment-free methods for detecting lateral genetic transfer and for classifying metagenomic data, to highlight further the power of alignment-free approaches.*

I also would have liked an expansion of the discussion of the problems created through multiple sequence alignments for downstream analyses. Ever since I attempted to analyse the evolutionary history of ATPase proteolipids [25], I am aware of the problems that multiple sequence alignments can create for phylogenetic analyses of divergent sequences, and I became a big fan of Thorne and Kishino’s approach to calculate phylogenies from pairwise sequence alignments [27]. While this approach certainly is not faster than ones based on MSAs, it avoids the bias created in MSAs, and provides conservative reliability estimates. Phylogenetic approaches that link sequence alignment to phylogenetic reconstruction, such as SATé [28] and Dactal [29], may be able to solve some of the MSA associated problems; however, as pointed out in the manuscript, approaches that are based on pairwise distances between sequences [32,37,47] calculated without a global alignment promise a faster and possibly equally reliable alternative.

**Authors’ response:***These issues are exactly why we think approaches independent of MSA could be a good strategy in next-generation phylogenomics. Approaches integrating MSA with phylogenetic reconstruction were proposed back in the 1980s*[48]*and 1990s*[49-51]*, but these methods are not scalable due to NP-hardness, e.g.*[52]*, and remain inevitably limited by the MSA framework. A thorough description of issues associated with MSA deserves a paper on its own*[21-23]*and is beyond the scope and limit of this Comment. We have expanded the text to highlight other methods developed to address some of the limitations of MSA, and how alignment-free methods could be an attractive alternative.*
